# Clinical characteristics of patients with tinnitus evaluated with the Tinnitus Sample Case History Questionnaire in Japan: A case series

**DOI:** 10.1371/journal.pone.0180609

**Published:** 2017-08-25

**Authors:** Takashi Kojima, Sho Kanzaki, Naoki Oishi, Kaoru Ogawa

**Affiliations:** 1 Department of Otolaryngology, Head and Neck Surgery, Keio University School of Medicine, Shinjuku-ku, Tokyo, Japan; 2 Department of Otorhinolaryngology, Machida Municipal Hospital, Machida-shi, Tokyo, Japan; University of Regensburg, GERMANY

## Abstract

**Background:**

The Tinnitus Sample Case History Questionnaire was determined as a standardized questionnaire for obtaining patient case histories and for characterizing patients into subgroups at the Tinnitus Research Initiative in 2006. In this study, we developed a Japanese version of this questionnaire for evaluating the clinical characteristics of patients with tinnitus. The Japanese version of the questionnaire will be available for evaluating treatments for tinnitus and for comparing data on tinnitus in research centers.

**Aims/Objectives:**

To evaluate the clinical characteristics of patients with tinnitus in Japan using a newly developed Japanese version of Tinnitus Sample Case History Questionnaire.

**Study design:**

This was a prospective study based on patient records.

**Setting:**

University hospitals, general hospitals, and clinics.

**Subjects and methods:**

We collected patient data using a Japanese translated version of the Tinnitus Sample Case History Questionnaire. In total, 584 patients who visited our institutions in Japan between August 2012 and March 2014 were included (280 males and 304 females; age 13–92 years; mean age, 60.8). We examined patients after dividing them into two groups according to the presence or absence of hyperacusis. The collected results were compared with those from the Tinnitus Research Initiative database.

**Results:**

Compared with the TRI database, there were significantly more elderly female patients and fewer patients with trauma-associated tinnitus. There was a statistically lower ratio of patients with hyperacusis. We found that patients with tinnitus in addition to hyperacusis had greater tinnitus severity and exhibited higher rates of various complications.

**Conclusion:**

The Japanese version of the Tinnitus Sample Case History Questionnaire developed in this study can be a useful tool for evaluating patients with tinnitus in Japan. The results of this multicenter study reflect the characteristics of patients with tinnitus who require medical care in Japan. Our data provides a preliminary basis for an international comparison of tinnitus epidemiology.

## Introduction

Chronic tinnitus, the perception of sound in the absence of an acoustic external stimulus, affects 5%–15% of the total population [1, 2]. It can be a debilitating and life-altering experience [1, 2]. A clinical practice guideline for tinnitus was published recently [3]. Despite a large number of epidemiologic studies conducted in several countries [2, 4–6], no well-established specific treatment is available for tinnitus. One of the difficulties is that it is unknown which subgroups of patients with tinnitus may benefit most from any particular treatment method. Although no standardized protocol has been established in Japan, we usually manage patients with tinnitus by following strategy. At first, we take a case history by an interview and evaluate auditory function. Then, we assess tinnitus severity by Tinnitus Handicap Inventory [7, 8], and psychiatric condition by some questionnaires (e.g. Hospital Anxiety and Depression Scale [9]). Based on these results, we plan management for each. Case histories may potentially classify patients into a particular subgroup, however; there is no standardized questionnaire. The different forms of tinnitus may be grouped into subtypes based on patient data, including etiological factors, clinical appearances, and comorbid medical conditions [10–13]. Among the reasons for the current lack of a satisfactory classification system is that tinnitus is an entirely self-reported subjective phenomenon.

It has become necessary to have a standard set of assessment methods to be widely agreed upon by the international tinnitus research community. An attempt was made to gain consensus on assessment tools at the Tinnitus Research Initiative (TRI) in Regensburg, Germany in 2006. The Tinnitus Sample Case History Questionnaire (TSCHQ) was developed as a standardized instrument for obtaining patients case histories and characterizing the disorder into subgroups [14]. The TRI has also established a new international tinnitus database [15] to collect and analyze data from around the world. TSCHQ has a predominance collecting patient data efficiently, including age, gender, tinnitus severity, and related comorbidities. Each item can be a basis for subgrouping. Moreover, the questionnaire takes unusual but tinnitus related symptom into account, such as hyperacusis. Hyperacusis is an auditory disorder characterized by a hypersensitivity or intolerance to external sound [16]. Although a large overlap between tinnitus and hyperacusis was suggested [16–18], an interrelationship and an etiology are still under the debate. An analysis for subgrouping may contribute to comprehending characteristics of both two disorders. A limitation of the current TRI database is that the data has been accumulated from predominantly Western countries. To achieve a global database reflecting characteristics of tinnitus patients worldwide, it is important to also accumulate data from other countries. As yet, there are no such repositories of information from Asia, the Middle East, or Africa. Institutions in these regions should also contribute to the TRI database. However, in order to do so, the TSCHQ needs to be universally available, first translated and adapted and then tested to demonstrate its validity and reliability for use in different cultures and in various languages.

The aim of this study was to develop a Japanese version of the TSCHQ to evaluate the clinical characteristics of patients with tinnitus in Japan prospectively. By comparing the results of our study with published data from the TRI database, we sought to demonstrate the efficacy of the translated TSCHQ in Japan. Also, we discussed the suitability of hyperacusis for subgrouping tinnitus.

## Materials and methods

### Validated translation

Validated translation of the TSCHQ was carried out in accordance with the methods outlined in the TRI database [15], and in addition to that, steps were taken in order to acquire cross-cultural adaptation. Step I, forward translation: two professional translators who were native Japanese speakers and fluent in English independently translated the original version of the TSCHQ into Japanese. Step II, confirmation of consistency: the two translators checked each other’s drafts and confirmed consistency. Step III, back-translation: these two translators converted the draft into English again and confirmed validity. Step IV, the expert committee: this committee was composed of seven otolaryngologists specializing in otology and audiology. The committee reviewed all translated items and confirmed consensus. Thirty-five items were translatable (S1 Appendix).

### Study design and patients

This prospective study included patients with subjective tinnitus examined at the otolaryngology departments of two university hospitals, five general hospitals, or six private otorhinolaryngology clinics between August 2012 and March 2014 in Japan. We provided the Japanese version of the TSCHQ to patients with tinnitus when they first presented with the chief complaint of tinnitus. Patients answered the questionnaire while waiting to be seen. Questionnaires were collected and analyzed at the Keio University Hospital.

Detail of this clinical research was displayed at a consultation room and oral consent was collected from all participants. According to the Japan Ethical Guidelines for Medical and Health Research Involving Human Subjects, we were not necessarily required to obtain informed consent. However, we notified the research subjects, or made public, information concerning the research including the purpose of collection and utilization of research information, and that there is an opportunity to refuse participation or remove their data from the study after commencement. This information was also documented in each patient’s chart. For all participants who were under 20 years of age, we received consent from the parents or legal guardians. The study was approved by the ethics committee of the Keio University School of Medicine (JPRN-UMIN000008901) and conducted in accordance with the Declaration of Helsinki. All patients consented to use of their data for future studies. Data were anonymized at the time of collection.

### Subgrouping

In order to classify subgroups of tinnitus, patient background, auditory data, modifying influences or comorbid symptoms could be categorizing factors. To compare with published data [18], we focused on hyperacusis in this study. Hyperacusis was defined by item 29 of the TSCHQ: “Do sounds cause you pain or physical discomfort?” We compared data between tinnitus patients who answered “Yes” and those who answered “No.”

### Analysis

We compared our data with the results published in the TRI database [10, 18–20] regarding features of patients with tinnitus (gender, tinnitus duration, onset related events, site of tinnitus and prevalence of hyperacusis). We divided the patients into two subgroups: those with and without hyperacusis. The chi-square test was used for categorical variables (referring hospital, gender, family history, onset style, onset related events, pulsatile, site of tinnitus, manifestation over time, fluctuating, tinnitus characteristics, subjective tinnitus pitch, treatments, maskability, aggravation by noise, somatic modulation, influence by nap, sleep, stress, hearing impairment, hearing aid, noise intolerance, headache, vertigo, temporomandibular joint, neck pain, other pain, and psychiatric problem), and two-sided t-tests for continuous variables (age, tinnitus duration, and tinnitus severity evaluating with following items in TSCHQ: subjective tinnitus loudness, subjective tinnitus awareness time, and subjective tinnitus annoyance). The significance threshold was set at <0.05. We reported the effect size using Cohen’s d. To maintain uniformity, the chi-square Pearson r coefficient was transformed into Cohen’s d effect size using the statistical calculation spreadsheet “Converting effect sizes” available at http://www.stat-help.com/, which is based on a published conversion formula [21]. Effect sizes were considered negligible with values <0.2, small with values ranging from 0.2 to 0.5, medium with values between 0.5 and 0.8, and large with values >0.8. We presented results calculated as significant (*p* <0.05) with at least a small effect size, only when these effects are considered clinically important. Statistical analysis was performed with SPSS Statistics software version 22 (IBM Corporation).

## Results

The overall results from the total sample of 584 patients who completed the Japanese version of the TSCHQ are shown in Table 1. Not every patient answered every question. Excluding two items with open-ended responses, the results list 34 items, including the type of medical facility. Some items permitted multiple responses, and only results with a single response were tabulated. Age at the visit was 13–92 years (median 63 years), and there were 304 females and 280 male participants. Mean tinnitus duration was 80.9 ± 132.9. For gender, our results included more women with tinnitus than the TRI database (*p* <0.001). The proportion of patients with trauma-associated tinnitus and hyperacusis in our study was smaller than the TRI database (*p* <0.001).

**Table 1:** Overall our results compared with data from the TRI database.

	Our study	Results from TRI	Statistics (x^2^; *p-*value)
**Background**			
Referring hospital (university/general hospital/clinic)	118/330/136 (n = 584); 20%/57%/23%		
1. Age at visit (years); median (IQR)	63 (51, 72) (n = 580)	52 (43, 61) (n = 1274)	
2. Gender (female/male)	304/280 (n = 584); 52%/48%	474/844 (n = 1318); 36%/64%	43.3; <0.001*
3. Handedness (right/left/both)	530/26/21 (n = 577); 92%/5%/4%		
4. Family history (no/yes)	434/104 (n = 538); 81%/19%		
**Tinnitus history**			
5. Tinnitus duration (mouths): mean; median; IQR	80.9 ± 132.9; 24; 46 (n = 518)	60.0; 19.2; 142.8 (n = 1274)	
6. Onset style (gradual/abrupt)	240/309 (n = 549); 43%/56%		
7. Onset related events (change in hearing/stress/trauma/others)	116/164/41/116/ (n = 472); 25%/35%/8%/25%/	327/565/567/573 (n = 1318); 25%/43%/43%/43%	83.9; <0.001*
8. Pulsatile (no/yes)	368/169 (n = 537); 69%/31%		
9. Site of tinnitus (right/left/both or within head)	144/192/313 (n = 649); 22%/30%/48%	391/504/401(n = 1318); 30%/38%/30%	55.7; <0.001*
10. Manifestation over time (intermittent/constant)	119/434 (n = 553); 21%/77%		
11. Fluctuating (no/yes)	252/314 (n = 566); 44%/55%		
12. Subjective tinnitus loudness (0–100)	56.0 ± 31.2 (n = 533)		
14. Character of tinnitus sound (tone/noise/crickets/others)	161/181/204/47 (n = 593); 27%/31%/34%/8%		
15. Subjective tinnitus pitch (very high/high/medium/low)	85/195/207/133 (n = 620); 14%/32%/33%/22%		
16. Subjective tinnitus awareness time (0–100)	67.1 ± 34.7 (n = 543)		
17. Subjective tinnitus annoyance (0–100)	46.5 ± 33.5 (n = 524)		
18Treatments (many/several/one/none)	14/93/150/288 (n = 545); 3%/17%/28%/53%		
**Modifying influences**			
19. Maskable by music or sound (no/yes/I don't know)	123/243/195 (n = 561); 43%/22%/35%		
20. Aggravated by noise (no/yes/ I don't know)	239/99/227 (n = 565); 42%/18%/40%		
21. Somatic modulation (no/yes)	468/85 (n = 553); 85%/15%		
22. Influence by nap (worsens/reduces/no effect)	32/56/423 (n = 511); 6%/11%/83%		
23. Influence by sleep (no/yes/I don't know)	156/92/310 (n = 558); 28%/17%/56%		
24. Influence by stress (worsen/reduces/no effect)	220/17/269 (n = 506); 44%/3%/53%		
21. Somatic modulation (no/yes)	42/21 (n = 63); 67%/33%		
**Related conditions**			
26. Hearing impairment (no/yes)	231/319 (n = 550); 42%/58%		
27. Hearing aid (no/yes)	529/38 (n = 567); 93%/7%		
28. Noise intolerance (never/rarely/sometime/usually/always)	333/73/101/27/12 (n = 546); 61%/13%/19%/5%/2%		
29. Hyperacusis (no/yes/I don't know)	363/118/86 (n = 567);64%/21%/15%	935/778/187 (n = 1900); 49%/41%/10%	78.1; <0.001*
30. Headache (no/yes)	389/177 (n = 566); 695/31%		
31. Vertigo or dizziness (no/yes)	350/213 (n = 563); 62%/38%		
32. Temporomandibular joint complaints (no/yes)	445/90 (n = 535); 835/17%		
33. Neck pain (no/yes)	374/188 (n = 562); 67%/34%		
34. Other pain (no/yes)	431/121 (n = 552); 78%/22%		
35. Psychiatric problems (no/yes)	512/57 (n = 569); 90%/10%		

Overall results of the Japanese version of Tinnitus Sample Case History Questionnaire and referring institutions compared with Published data from TRI database. Our results included significantly more women with tinnitus than the TRI database. The distribution of patients with trauma-associated tinnitus and hyperacusis in our study was smaller than the TRI database.

* *p* <0.05, statistically significant (chi-square test)

Of 567 patients who responded to the question about hyperacusis, 118 (21%) said ‘yes,’ 363 (64%) said ‘no,’ and the remaining 86 said that they did not know. The comparison of those with and without hyperacusis is shown in Table 2. Patients with hyperacusis were significantly more likely to visit university hospitals (*p* = 0.004, d = 0.31). There was no significant difference between hyperacusis vs. non-hyperacusis group for age (*p* = 0.653, d = 0.04) or gender (*p* = 0.214, d = 0.11), although tinnitus patients with hyperacusis were significantly younger and there were more female patients in the TRI database [18]. Schecklmann also showed individuals with hyperacusis were more likely to have tinnitus which was more severe, pulsatile in nature, made worsen by somatic modulation, influenced by noise, sleep, and stress, severer tinnitus, and more likely to suffer from comorbidities [18]. Pulsatile tinnitus (*p* = 0.003, d = 0.28) was significantly more common among those with hyperacusis, but the effect size was small. Patients with hyperacusis were significantly more likely to have difficulty tolerating sound symptoms (*p* <0.001, d = 1.10) and to recognize the influence of noise (*p* <0.001, d = 0.32), stress (*p* <0.001, d = 0.50), sleep (*p* = 0.014, d = 0.01), and naps (*p* = 0.003, d = 0.32) on their symptoms. Patients with hyperacusis complained significantly had greater tinnitus severity (*p* <0.001, d = 0.40), annoyance (*p* <0.001, d = 0.56), and hearing problems (*p* <0.001, d = 0.45). They also had significantly more medical comorbidities.

**Table 2:** Characteristics of patients with tinnitus and with or without hyperacusis.

	Tinnitus (n = 363)	Tinnitus with hyperacusis (n = 118)	Statistics (t/x^2^; *p*-value; Cohen d)
**Background**			
**Referring hospital (university/general hospital/clinic)**	**65/195/103; 18%/54%/28%**	**35/64/19; 30%/54%/16%**	**11.21; 0.004; 0.31**
1. Age at visit (years)	61.0 ± 15.8	60.4 ± 15.9	−0.358; 0.653; 0.04
2. Gender (female/male)	190/173; 52%/48%	54/64; 46%/54%	1.54; 0.214; 0.11
3. Handedness (right/left/both)	330/14/14;92%/4%/4%	104/9/5;88%/8%/4%	2.72; 0.28; 0.15
**Tinnitus history**			
5. Tinnitus duration (mouths)	82.9 ± 136.1	84.7 ± 136.8	0.12; 0.90; 0.01
6. Onset style (gradual/abrupt)	156/185; 46%/54%	47/68; 59%/41%	0.83; 0.36; 0.09
7. Onset related events (change in hearing/stress/trauma/others/none)	55/75/21/23; 32%/43%/12%/13%	21/31/7/6; 32%/48%/11%/9%	0.92; 0.82; 0.11
**8. Pulsatile (no/yes)**	**244/92; 73%/27%**	**64/47; 58%/42%**	**8.72; 0.003; 0.28**
9. Site of tinnitus (right/left/both or within head)	88/96/102/29/6; 27%/30%/32%/9%/2%	25/32/35/9/1; 25%/31%/34%/9%/1%	0.83; 0.94; 0.00
10. Manifestation over time (intermittent/constant)	77/268; 22%/78%	20/92; 18%/82%	1.01; 0.32; 0.09
11. Fluctuating (no/yes)	169/187; 47%/53%	48/69; 41%/59%	1.42; 0.49; 0.10
**12. Subjective tinnitus loudness (0–100)**	**52.0 ± 30.7**	**64.1 ± 30.1**	**3.63; <0.001; 0.40**
14. Character of tinnitus sound (tone/noise/crickets/others)	96/79/106/27; 31%/26%/34%/9%	22/32/25/11; 24%/36%/28%/12%	5.32; 0.15; 0.63
15. Subjective tinnitus pitch (very high/high/medium/low)	47/91/111/71; 15%/28%/35%/22%	13/44/34/13; 13%/42%/33%/13%	8.82; 0.03; 0.17
16. Subjective tinnitus awareness time (0–100)	65.4 ± 35.4	71.0 **±** 33.5	1.49; 0.14; 0.16
**17. Subjective tinnitus annoyance (0–100)**	**40.0 ± 31.9**	**57.9 ± 33.2**	**5.03; <0.001; 0.56**
**18. Treatments (many/** **several/one/none)**	**5/56/84/197; 2%/16%/25%/58%**	**7/27/31/45; 6%/25%/28%/41%**	**15.32; 0.002; 0.35**
**Modifying influences**			
19. Maskable by music or sound (no/yes/I don't know)	76/163/113; 22%/46%/32%	31/47/37; 27%/41%/32%	1.66; 0.44; 0.05
**20. Aggravated by noise (no/yes/I don't know)**	**183/41/133; 51%/12%/37%**	**30/39/46; 26%/34%/40%**	**38.20; <0.001; 0.24**
21. Somatic modulation (no/yes)	296/52; 85%/15%	93/20; 82%/18%	0.49; 0.48; 0.07
**22. Influence by nap (worsens/reduces/no effect)**	**14/29/285;4%/9%/87%**	**10/18/75; 10%/18%/73%**	**11.38; 0.003; 0.32**
23. Influence by sleep (no/yes/I don't know)	120/53/177;34%/15%/51%	23/25/64; 21%/22%/57%	8.48; 0.014; 0.01
**24. Influence by stress (worsen/reduces/no effect)**	**110/13/199;34%/4%/62%**	**65/1/37;63%/1%/36%**	**27.52; <0.001; 0.50**
**Related conditions**			
**26. Hearing impairment (no/yes)**	**166/182; 48%/52%**	**26/88; 23%/77%**	**21.91; <0.001; 0.45**
27. Hearing aid (no/yes)	336/23; 94%/6%	107/8; 93%/7%	0.04; 0.84; 0.002
**28. Noise intolerance (never/rarely/sometime/usually/always)**	**261/42/42/3/3;74%/12%/12/%1%/1%**	**32/13/40/17/9;29%/12%36%/15%/8%**	**112.9; <0.001; 1.10**
**30. Headache (no/yes)**	**263/96; 73%/27%**	**72/45; 62%/39%**	**5.82; 0.016; 0.22**
**31. Vertigo or dizziness (no/yes)**	**245/116; 68%/32%**	**63/53; 54%/46%**	**7.05; 0.008; 0.25**
32. Temporomandibular joint complaints (no/yes)	292/53; 85%/15%	86/26; 77%/23%	3.65; 0.056; 0.18
**33. Neck pain (no/yes)**	**252/106; 70%/30%**	**62/55; 53%/47%**	**11.92; 0.001; 0.32**
**34. Other pain (no/yes)**	**288/65; 82%/18%**	**80/36; 69%/31%**	**8.23; 0.004; 0.27**
**35. Psychiatric problems (no/yes)**	**336/27; 93%/7%**	**96/22; 81%/19%**	**12.22; <0.001; 0.32**

Patients with hyperacusis complained more severe tinnitus-related symptom than those without hyperacusis; including subjective tinnitus loudness and annoyance evaluating in this questionnaire, hearing impairment, a headache, vertigo, neck pain, other pain, and psychiatric problem. Meaningful contrasts are defined by *p* <0.05 and d >0.2 and marked in bold font.

## Discussion

To our knowledge, this is the first report on collecting data about patients with tinnitus using the TSCHQ in an Asian country. The data collected in the present study were obtained from various hospitals and therefore reflect the characteristics of tinnitus patients throughout Japan. Patients with hyperacusis complained of severe tinnitus from the aspects of subjective tinnitus loudness and annoyance evaluating in this questionnaire, hearing impairment, and various comorbid symptoms, including a headache, vertigo, neck pain, other pain and psychiatric problem.

Several studies have been performed to analyze different treatment efficacies for patients with tinnitus [22–24]. However, comparison of each study is difficult, because each research developed its own measurement system as an assessment tool. To compare future studies with greater validity, the TRI database focused on homogenization of patient backgrounds and attempted to construct a cross-national database [14, 15]. Rational, cultural or ethical discrepancies have been shown to influence pure-tone thresholds [25] as well as the psychiatric phenotype of patients with depression [26]. These discrepancies may also generate differences in tinnitus patient histories. Therefore, our result can contribute to future tinnitus research in terms of demonstration of patient characteristics and background in Asian countries.

Patients with tinnitus in this study were older and included more women than the TRI database. There was a low incidence of trauma-associated tinnitus, with stress being the most common onset-related event in Japan. Although there is likely to be a commonly accepted relationship between stress and tinnitus, clinical evidence is still insufficient [27]. Stress was sometimes reported as a possible cause of tinnitus [10, 28], and the degree of tinnitus distress showed a multidimensional relationship to hearing impairment [29], somatic discomfort [12] and depressive mood [30]. This questionnaire has a clinical advantage to be able to categorize patients whose tinnitus is caused by stress. The reason of the highest distribution of stress related tinnitus was unclear; therefore, further study is required. There may also be some overlap between stress and a change in hearing, as this in itself could be considered stressful to some degree by some patients [28, 30]. As many studies have revealed, hearing loss associated with tinnitus [3, 29, 31, 32]. Therefore, we suppose ‘change in hearing’ involves hearing loss or obstruction of the hearing pathways. The reason for these differences between the TRI data and ours is unclear. They may reflect social or regional differences. Further accumulation of data is required to explore these findings. We found that 58% of patients with tinnitus experienced subjective hearing problems. A change in hearing was the onset-related event in 25% of patients and was not necessarily consistent. We believe that tinnitus may be triggered by stress in some patients who already had a hearing impairment before the onset of tinnitus. Compared with the reported rate of hearing disorders, there was an extremely low rate of hearing aid use. Several reports have indicated that acoustic therapy with a hearing aid is an effective treatment for tinnitus [33]. Therefore, it would be worth trying a hearing aid to see if it would effectively manage the tinnitus.

The large overlap in the prevalence of tinnitus and hyperacusis suggests that the two disorders share common pathophysiologic mechanisms and risk factors [16]. The prevalence of hyperacusis in patients in our study was lower than that in the TRI database [18] and described in a previous report [16]. Furthermore, while reports based on the TRI database indicate that subjects with hyperacusis mainly included young men, in the present study, we found no age- or sex-related differences. Again, if most patients who experience hyperacusis are young [18], the low prevalence in our study may be because our patients were relatively older and included more women. We also found that an inability to tolerate sound had the largest effect in patients with hyperacusis. Some reports indicate that a presence of a common neurophysiological pathway that contributes to an etiology of tinnitus and hyperacusis [28, 32]; therefore, the issue of noise intolerance may be similar in tinnitus and hyperacusis. In addition, patients with hyperacusis had more severe symptoms, including subjective tinnitus annoyance, loudness, experiences for tinnitus treatment, and related comorbidities. Thus, tinnitus with hyperacusis is a serious condition that may require more intensive treatment. For example, we not only recommend a general remedy to the patient with hyperacusis but also encourage them to treat their comorbid conditions and to visit a psychiatrist if they are with psychological symptoms. The association between naps and hyperacusis remains unclear; if this phenomenon is to be understood, further studies will be required. We found that certain clinical features of patients with tinnitus differ between those who do or do not have hyperacusis; therefore, identifying patients with both tinnitus and hyperacusis may be useful from a treatment perspective.

Translations of questionnaires into another language may be incorrect, so that participants may misinterpret the intent of the questions. In the present study, we experienced several difficulties when translating the TSCHQ into Japanese, including problems with onomatopoeia and ambiguous expressions such as “frequency.” More precisely, these issues corresponded to descriptions of sound, such as “tone,” “crickets,” and “noise,” in item 14 and expressions of frequency, such as “several,” “many,” “often,” “sometimes,” and “usually” in item 28. These terms have no direct equivalent in Japanese or are difficult to understand intuitively and may, therefore, have impeded the overall data collection in the present study. Measures must be taken so that ambiguous expressions are not included in the study. It would be better if values could be expressed in real numbers rather than in vague frequencies.

### Conclusions

This is the first study to collect patient case histories with a translated TSCHQ in an Asian country. The Japanese translation of the TSCHQ was entirely feasible for use in Japan and enabled valid data collection. We believe that the differences we found in our results compared with the data obtained from the TRI database may have arisen from regional or cultural differences. Because this was a preliminary study, further case accumulation is needed. To further understand the clinical properties of tinnitus according to subgroups, we recommend the creation of a global database.

## Supporting information

S1 Appendix Japanese version of Tinnitus Sample Case History Questionnaire. (PDF)

S1 Table Original data of Japanese version of Tinnitus Sample Case History Questionnaire. (XLSX)
