# Supplementary material for: Clinical characteristics of patients with tinnitus evaluated with the Tinnitus Sample Case History Questionnaire in Japan: A case series
Source: PLoS One. 2017 Aug 25;12(8):e0180609. doi: 10.1371/journal.pone.0180609 (PMC5571926; doi:10.1371/journal.pone.0180609)
Supplement: S1 Appendix — (PDF) [file pone.0180609.s001.pdf]

1. 年齢
2. 性別：男性　女性
3. 利き手：右利き　左利き　両利き
4. 耳鳴の家族歴：ある　（ある場合：両親　兄弟姉妹　子供）　ない
5. 最初の発症：耳鳴を最初に感じたのはいつですか？：____________
6. どのように始まりましたか？：徐々に　突然
7. 最初に感じた耳鳴に関係していたものはありますか？：爆発するような大きな音　むち打ち症　張力の悪化　ストレス　頭の怪我　その他____________
8. 耳鳴は脈打つように感じられますか？：はい、鼓動と同じリズム　はい、鼓動とは異なるリズム　いいえ
9. 耳鳴はどこで感じますか？：右耳　左耳　両耳だが左のほうがひどい　両耳だが右のほうがひどい　両耳で等しく　頭の中　その他__________
10. 耳鳴は時間的な現れ方はどのようですか？：間をおいて　持続的に
11. 耳鳴の大きさは日によって変わりますか？：はい　いいえ
12. 耳鳴の大きさを１～１００の段階で表してください（１＝非常にかすかに聞こえる．　１００＝非常に大きく聞こえる）：__________（１～１００のいずれか）
13. 耳鳴は通常とのように聞こえるか、ご自分の言葉で説明してください：__________

    以下に，聞こえる可能性のある音の例を挙げますが，他の表現も自由にお使いください：
    シューシュー（風を切る音），ベルの鳴るような音，ドクドク（脈打つ音），ブーン，カチッ，メリッ，ピポパ（ダイヤル音などのプッシュ信号），ブンブン（羽音など），ポン（はじける音），ゴーゴー（轟音），ザーザー（勢いよく流れる音），タイプライター音（カタカタ），ヒューヒュー，シュー
14. 耳鳴の音色は澄んだ音でしたか，それともどちらかと言うと雑音のような音でしたか？：澄んだ音（ピーなど）　雑音（ザーなど）　虫の鳴き声（ジーなど）　その他__________
15. 耳鳴の音の高さはどうですか？：非常に高い音（高い周波数）　高い音　中くらい　低い音（低い周波数）
16. 最近１ヶ月間で，起きている時間のうち，どのくらいの割合で耳鳴がしていましたか？（例えば起きている時間いつも気になっていた場合は１００％，起きていた時間の１／４程度の時間であれば２５％としてください．）：__________％（１～１００のいずれか）

17. 最近１ヶ月間で，起きている時間のうち，どのくらいの割合で耳鳴に悩まされたり，苦痛を感じたり，イライラしたりしましたか？（上記の設問と同様に１～１００でお答えください．）：___________％（１～１００のいずれか）
18. 今まで耳鳴に対して何種類の治療を受けられましたか？：なし　１種類　２～５種類　たくさん
19. 音楽や滝の音のような環境音，シャワーを浴びる時の水の音などで耳鳴は軽減されますか？：はい　いいえ　わからない
20. 大きな音を聞いていると耳鳴は悪化しますか：はい　いいえ　わからない
21. 頭や首を動かす（下顎を前に突き出す．歯を食いしばる），腕／手または頭に触れられるなどで耳鳴は変化しますか？：はい　いいえ
22. 昼寝をすると耳鳴は変化しますか？：耳鳴が悪化する　耳鳴が軽減する　影響なし
23. 夜間の睡眠と昼間の耳鳴に関係がありますか？：はい　いいえ　わからない
24. ストレスは耳鳴に影響しますか？：耳鳴が悪化　耳鳴が軽減　影響なし
25. 耳鳴に影響するような薬はありますか？：薬剤名__________________　影響とその具体的な内容__________________
26. 聞こえ方について師匠を感じますか？：はい　いいえ
27. 補聴器を使用していますか？：右耳　左耳　両耳　使用していない
28. 周囲の人にとっては快適と思われる音が，あなたにとっては音が大きすぎて耐えられないものに感じることがありますか？：ない　まれにある　時々ある　よくある　いつも
29. 音を聞くことで痛みを感じたり，身体的な不快感を感じることがありますか？：はい　いいえ　わからない
30. 頭痛はありますか？：はい　いいえ
31. めまいやふらつき感はありますか？：はい　いいえ
32. 顎関節症などかみ合わせに問題はありますか？：はい　いいえ
33. 首の痛みはありますか？：はい　いいえ
34. 他に痛みを伴う病気がありますか？：はい　いいえ
35. 現在，精神疾患治療を受けられたことはありますか？：はい　いいえ

Appendix 1. All items of Japanese version of TSHCQ.
